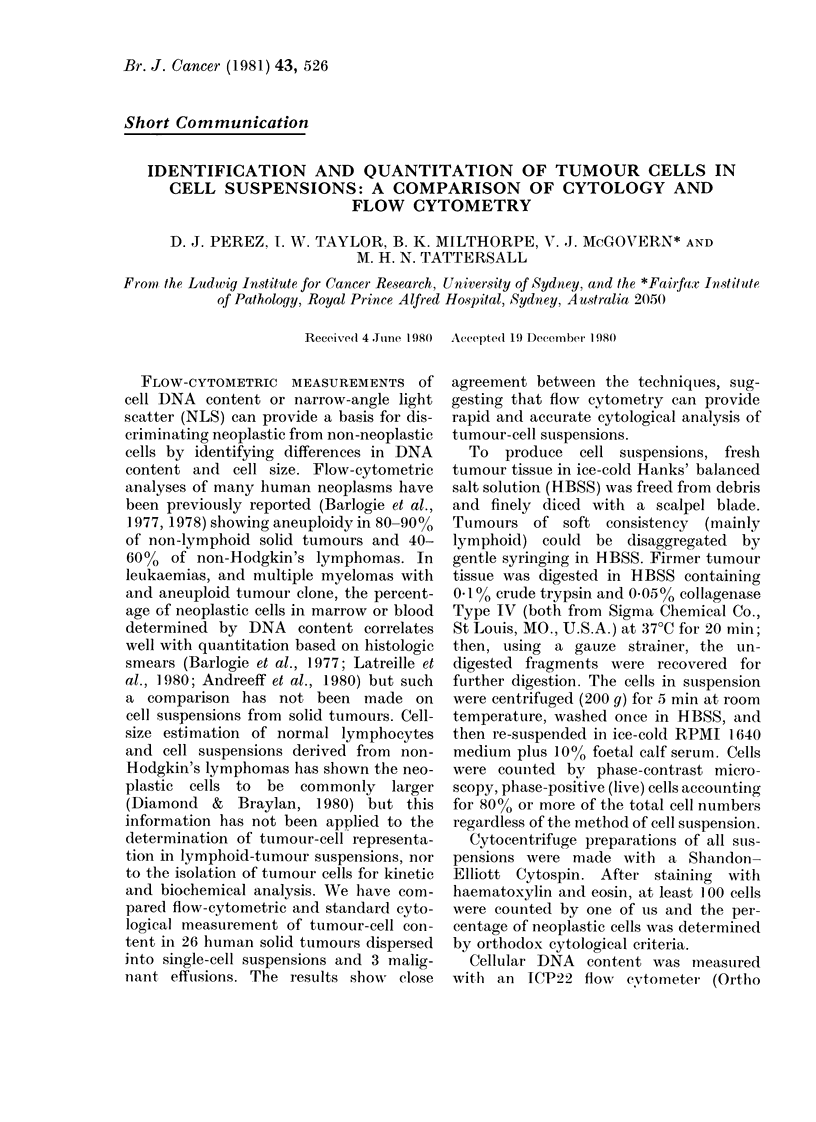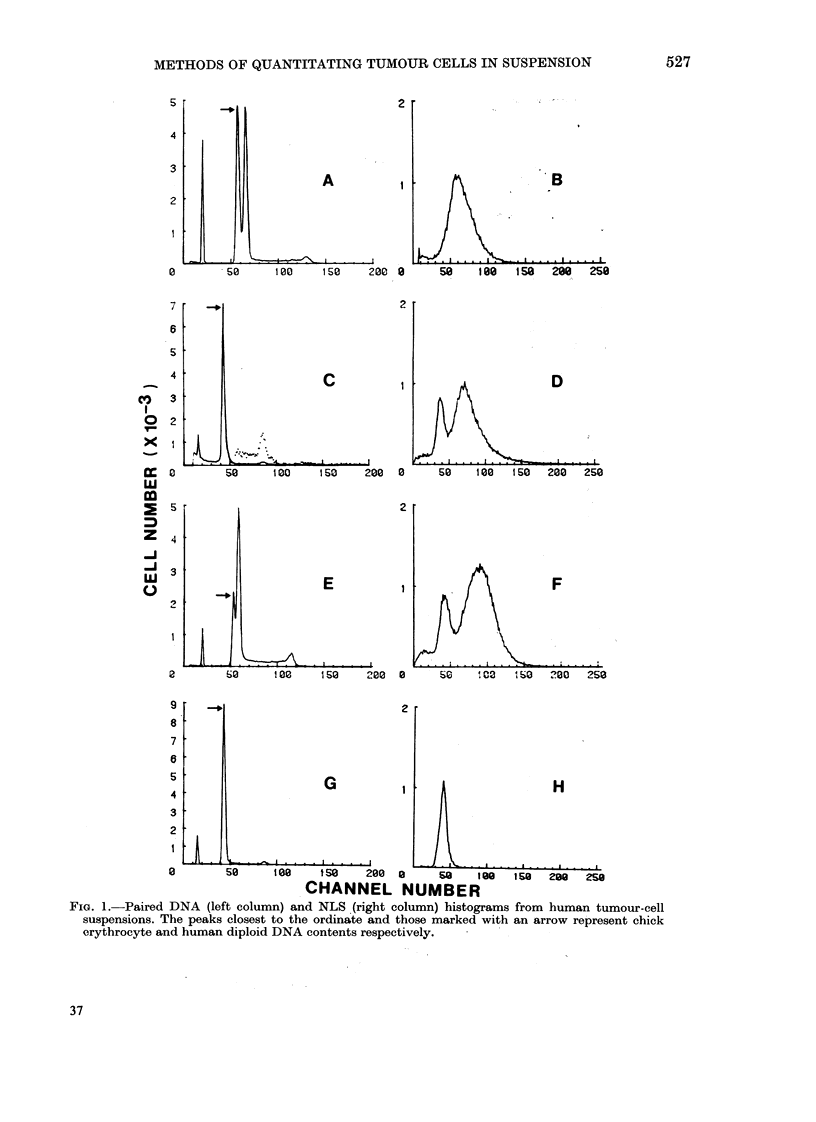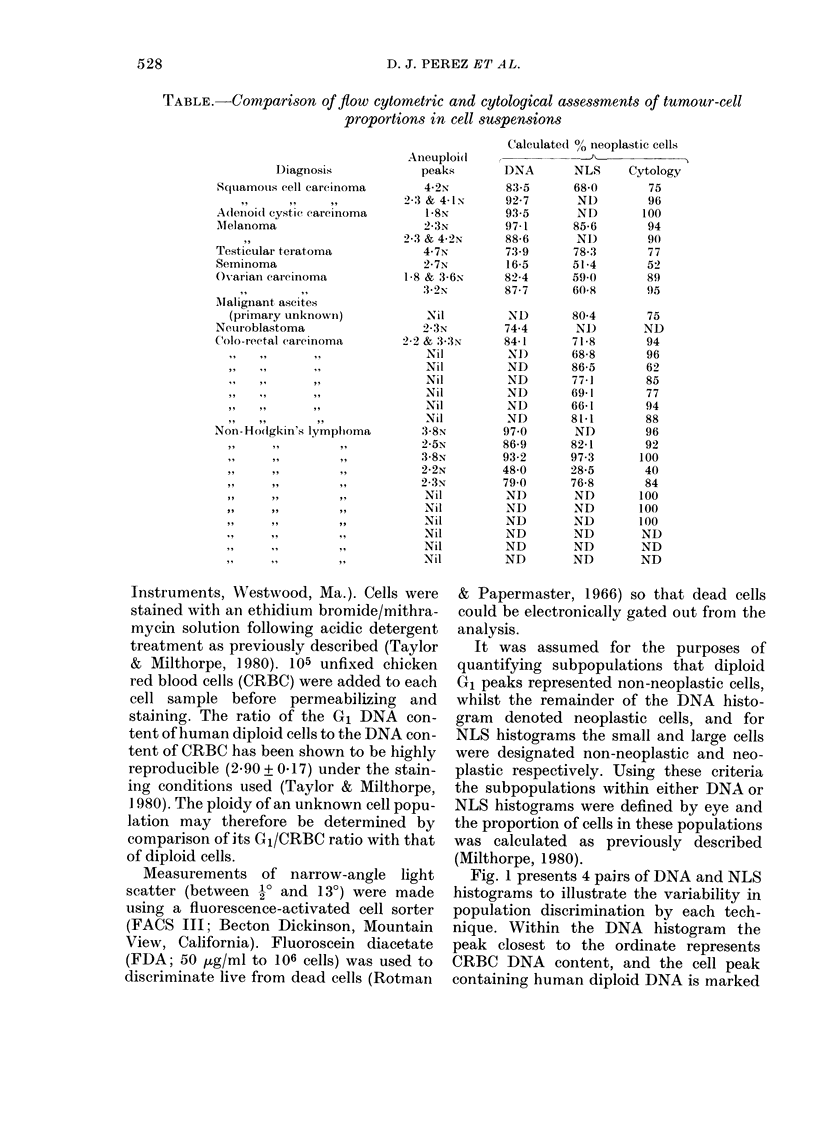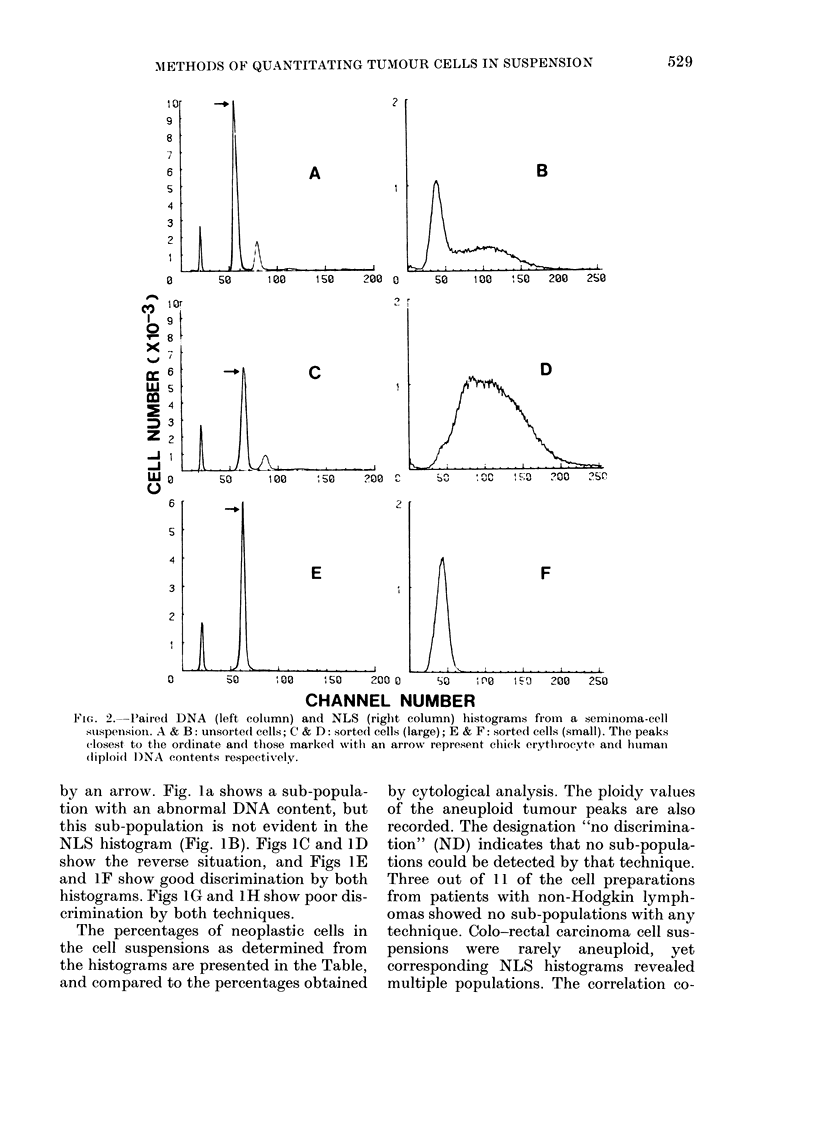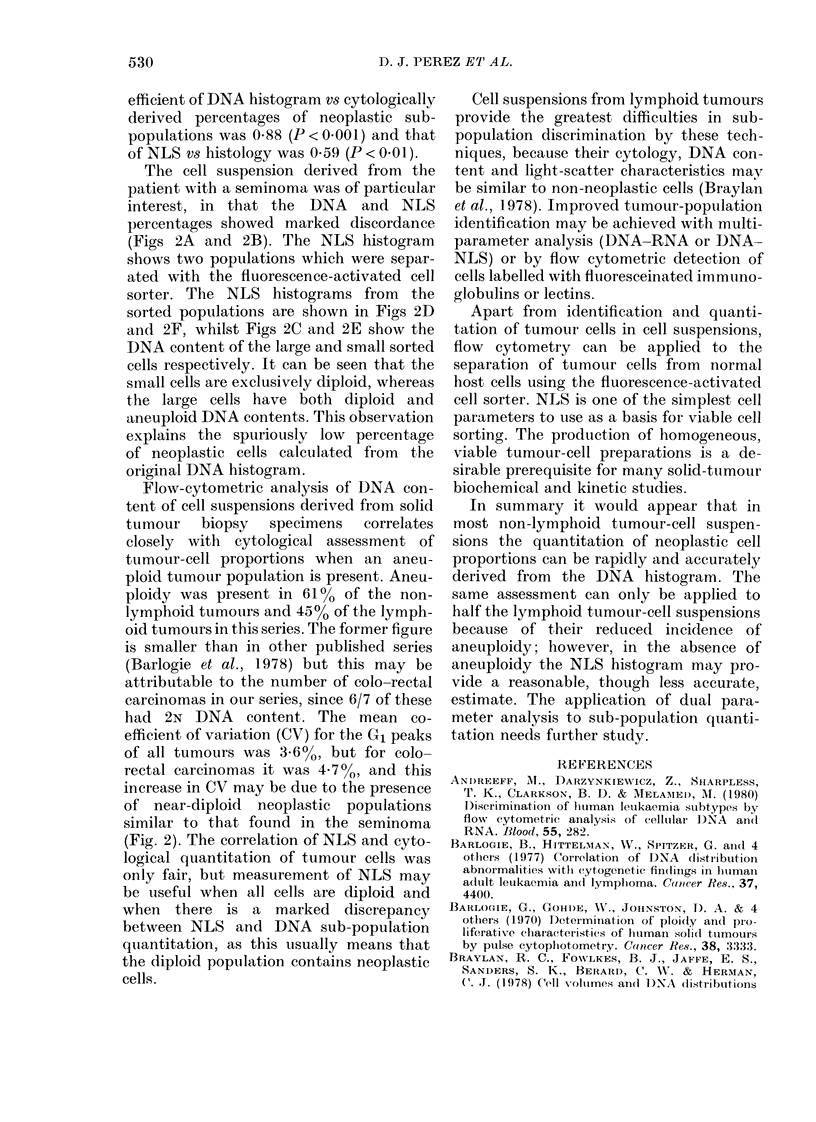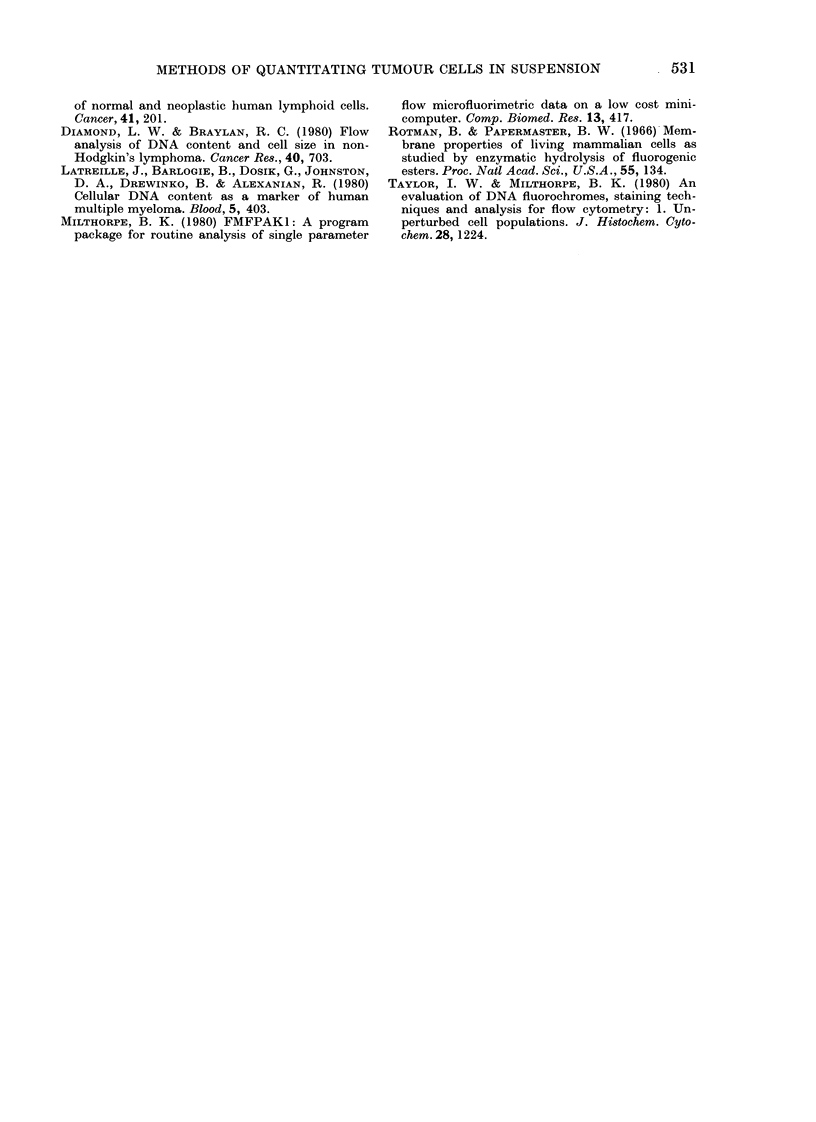# Identification and quantitation of tumour cells in cell suspensions: a comparison of cytology and flow cytometry.

**DOI:** 10.1038/bjc.1981.75

**Published:** 1981-04

**Authors:** D. J. Perez, I. W. Taylor, B. K. Milthorpe, V. J. McGovern, M. H. Tattersall


*Br. J. Cancer* (1981) **43**, 526

### *Short Communication*

### IDENTIFICATION AND QUANTITATION OF TUMOUR CELLS IN CELL SUSPENSIONS: A COMPARISON OF CYTOLOGY AND FLOW CYTOMETRY

D. J. PEREZ, I. W. TAYLOR, B. K. MILTHORPE, V. J. McGOVERN* AND M. H. N. TATTERSALL

*From the Ludwig Institute for Cancer Research, University of Sydney, and the *Fairfax Institute of Pathology, Royal Prince Alfred Hospital, Sydney, Australia* 2050

Received 4 June 1980 Accepted 19 December 1980

FLOW-CYTOMETRIC MEASUREMENTS of cell DNA content or narrow-angle light scatter (NLS) can provide a basis for discriminating neoplastic from non-neoplastic cells by identifying differences in DNA content and cell size. Flow-cytometric analyses of many human neoplasms have been previously reported (Barlogie *et al.*, 1977, 1978) showing aneuploidy in 80–90% of non-lymphoid solid tumours and 40–60% of non-Hodgkin's lymphomas. In leukaemias, and multiple myelomas with and aneuploid tumour clone, the percentage of neoplastic cells in marrow or blood determined by DNA content correlates well with quantitation based on histologic smears (Barlogie *et al.*, 1977; Latreille *et al.*, 1980; Andreeff *et al.*, 1980) but such a comparison has not been made on cell suspensions from solid tumours. Cell-size estimation of normal lymphocytes and cell suspensions derived from non-Hodgkin's lymphomas has shown the neoplastic cells to be commonly larger (Diamond & Braylan, 1980) but this information has not been applied to the determination of tumour-cell representation in lymphoid-tumour suspensions, nor to the isolation of tumour cells for kinetic and biochemical analysis. We have compared flow-cytometric and standard cytological measurement of tumour-cell content in 26 human solid tumours dispersed into single-cell suspensions and 3 malignant effusions. The results show close agreement between the techniques, suggesting that flow cytometry can provide rapid and accurate cytological analysis of tumour-cell suspensions.

To produce cell suspensions, fresh tumour tissue in ice-cold Hanks' balanced salt solution (HBSS) was freed from debris and finely diced with a scalpel blade. Tumours of soft consistency (mainly lymphoid) could be disaggregated by gentle syringing in HBSS. Firmer tumour tissue was digested in HBSS containing 0·1% crude trypsin and 0·05% collagenase Type IV (both from Sigma Chemical Co., St Louis, MO., U.S.A.) at 37°C for 20 min; then, using a gauze strainer, the undigested fragments were recovered for further digestion. The cells in suspension were centrifuged (200 *g*) for 5 min at room temperature, washed once in HBSS, and then re-suspended in ice-cold RPMI 1640 medium plus 10% foetal calf serum. Cells were counted by phase-contrast microscopy, phase-positive (live) cells accounting for 80% or more of the total cell numbers regardless of the method of cell suspension.

Cytocentrifuge preparations of all suspensions were made with a Shandon–Elliott Cytospin. After staining with haematoxylin and eosin, at least 100 cells were counted by one of us and the percentage of neoplastic cells was determined by orthodox cytological criteria.

Cellular DNA content was measured with an ICP22 flow cytometer (Ortho

FIG. 1.—Paired DNA (left column) and NLS (right column) histograms from human tumour-cell suspensions. The peaks closest to the ordinate and those marked with an arrow represent chick erythrocyte and human diploid DNA contents respectively.

TABLE.—*Comparison of flow cytometric and cytological assessments of tumour-cell proportions in cell suspensions*

| Diagnosis | Aneuploid peaks | Calculated % neoplastic cells DNA | NLS | Cytology |
|---|---|---|---|---|
| Squamous cell carcinoma | 4·2N | 83·5 | 68·0 | 75 |
| ,, ,, ,, | 2·3 & 4·1N | 92·7 | ND | 96 |
| Adenoid cystic carcinoma | 1·8N | 93·5 | ND | 100 |
| Melanoma | 2·3N | 97·1 | 85·6 | 94 |
| ,, | 2·3 & 4·2N | 88·6 | ND | 90 |
| Testicular teratoma | 4·7N | 73·9 | 78·3 | 77 |
| Seminoma | 2·7N | 16·5 | 51·4 | 52 |
| Ovarian carcinoma | 1·8 & 3·6N | 82·4 | 59·0 | 89 |
| ,, ,, | 3·2N | 87·7 | 60·8 | 95 |
| Malignant ascites (primary unknown) | Nil | ND | 80·4 | 75 |
| Neuroblastoma | 2·3N | 74·4 | ND | ND |
| Colo-rectal carcinoma | 2·2 & 3·3N | 84·1 | 71·8 | 94 |
| ,, ,, ,, | Nil | ND | 68·8 | 96 |
| ,, ,, ,, | Nil | ND | 86·5 | 62 |
| ,, ,, ,, | Nil | ND | 77·1 | 85 |
| ,, ,, ,, | Nil | ND | 69·1 | 77 |
| ,, ,, ,, | Nil | ND | 66·1 | 94 |
| ,, ,, ,, | Nil | ND | 81·1 | 88 |
| Non-Hodgkin's lymphoma | 3·8N | 97·0 | ND | 96 |
| ,, ,, ,, | 2·5N | 86·9 | 82·1 | 92 |
| ,, ,, ,, | 3·8N | 93·2 | 97·3 | 100 |
| ,, ,, ,, | 2·2N | 48·0 | 28·5 | 40 |
| ,, ,, ,, | 2·3N | 79·0 | 76·8 | 84 |
| ,, ,, ,, | Nil | ND | ND | 100 |
| ,, ,, ,, | Nil | ND | ND | 100 |
| ,, ,, ,, | Nil | ND | ND | 100 |
| ,, ,, ,, | Nil | ND | ND | ND |
| ,, ,, ,, | Nil | ND | ND | ND |
| ,, ,, ,, | Nil | ND | ND | ND |

Instruments, Westwood, Ma.). Cells were stained with an ethidium bromide/mithramycin solution following acidic detergent treatment as previously described (Taylor & Milthorpe, 1980). $10^5$ unfixed chicken red blood cells (CRBC) were added to each cell sample before permeabilizing and staining. The ratio of the $G_1$ DNA content of human diploid cells to the DNA content of CRBC has been shown to be highly reproducible ($2·90 \pm 0·17$) under the staining conditions used (Taylor & Milthorpe, 1980). The ploidy of an unknown cell population may therefore be determined by comparison of its $G_1$/CRBC ratio with that of diploid cells.

Measurements of narrow-angle light scatter (between $\frac{1}{2}°$ and 13°) were made using a fluorescence-activated cell sorter (FACS III; Becton Dickinson, Mountain View, California). Fluoroscein diacetate (FDA; 50 $\mu$g/ml to $10^6$ cells) was used to discriminate live from dead cells (Rotman & Papermaster, 1966) so that dead cells could be electronically gated out from the analysis.

It was assumed for the purposes of quantifying subpopulations that diploid $G_1$ peaks represented non-neoplastic cells, whilst the remainder of the DNA histogram denoted neoplastic cells, and for NLS histograms the small and large cells were designated non-neoplastic and neoplastic respectively. Using these criteria the subpopulations within either DNA or NLS histograms were defined by eye and the proportion of cells in these populations was calculated as previously described (Milthorpe, 1980).

Fig. 1 presents 4 pairs of DNA and NLS histograms to illustrate the variability in population discrimination by each technique. Within the DNA histogram the peak closest to the ordinate represents CRBC DNA content, and the cell peak containing human diploid DNA is marked

FIG. 2.—Paired DNA (left column) and NLS (right column) histograms from a seminoma-cell suspension. A & B: unsorted cells; C & D: sorted cells (large); E & F: sorted cells (small). The peaks closest to the ordinate and those marked with an arrow represent chick erythrocyte and human diploid DNA contents respectively.

by an arrow. Fig. 1a shows a sub-population with an abnormal DNA content, but this sub-population is not evident in the NLS histogram (Fig. 1B). Figs 1C and 1D show the reverse situation, and Figs 1E and 1F show good discrimination by both histograms. Figs 1G and 1H show poor discrimination by both techniques.

The percentages of neoplastic cells in the cell suspensions as determined from the histograms are presented in the Table, and compared to the percentages obtained by cytological analysis. The ploidy values of the aneuploid tumour peaks are also recorded. The designation "no discrimination" (ND) indicates that no sub-populations could be detected by that technique. Three out of 11 of the cell preparations from patients with non-Hodgkin lymphomas showed no sub-populations with any technique. Colo–rectal carcinoma cell suspensions were rarely aneuploid, yet corresponding NLS histograms revealed multiple populations. The correlation coefficient of DNA histogram *vs* cytologically derived percentages of neoplastic sub-populations was 0·88 ($P < 0·001$) and that of NLS *vs* histology was 0·59 ($P < 0·01$).

The cell suspension derived from the patient with a seminoma was of particular interest, in that the DNA and NLS percentages showed marked discordance (Figs 2A and 2B). The NLS histogram shows two populations which were separated with the fluorescence-activated cell sorter. The NLS histograms from the sorted populations are shown in Figs 2D and 2F, whilst Figs 2C and 2E show the DNA content of the large and small sorted cells respectively. It can be seen that the small cells are exclusively diploid, whereas the large cells have both diploid and aneuploid DNA contents. This observation explains the spuriously low percentage of neoplastic cells calculated from the original DNA histogram.

Flow-cytometric analysis of DNA content of cell suspensions derived from solid tumour biopsy specimens correlates closely with cytological assessment of tumour-cell proportions when an aneuploid tumour population is present. Aneuploidy was present in 61% of the non-lymphoid tumours and 45% of the lymphoid tumours in this series. The former figure is smaller than in other published series (Barlogie *et al.*, 1978) but this may be attributable to the number of colo–rectal carcinomas in our series, since 6/7 of these had 2N DNA content. The mean coefficient of variation (CV) for the $G_1$ peaks of all tumours was 3·6%, but for colo–rectal carcinomas it was 4·7%, and this increase in CV may be due to the presence of near-diploid neoplastic populations similar to that found in the seminoma (Fig. 2). The correlation of NLS and cytological quantitation of tumour cells was only fair, but measurement of NLS may be useful when all cells are diploid and when there is a marked discrepancy between NLS and DNA sub-population quantitation, as this usually means that the diploid population contains neoplastic cells.

Cell suspensions from lymphoid tumours provide the greatest difficulties in sub-population discrimination by these techniques, because their cytology, DNA content and light-scatter characteristics may be similar to non-neoplastic cells (Braylan *et al.*, 1978). Improved tumour-population identification may be achieved with multiparameter analysis (DNA–RNA or DNA–NLS) or by flow cytometric detection of cells labelled with fluoresceinated immunoglobulins or lectins.

Apart from identification and quantitation of tumour cells in cell suspensions, flow cytometry can be applied to the separation of tumour cells from normal host cells using the fluorescence-activated cell sorter. NLS is one of the simplest cell parameters to use as a basis for viable cell sorting. The production of homogeneous, viable tumour-cell preparations is a desirable prerequisite for many solid-tumour biochemical and kinetic studies.

In summary it would appear that in most non-lymphoid tumour-cell suspensions the quantitation of neoplastic cell proportions can be rapidly and accurately derived from the DNA histogram. The same assessment can only be applied to half the lymphoid tumour-cell suspensions because of their reduced incidence of aneuploidy; however, in the absence of aneuploidy the NLS histogram may provide a reasonable, though less accurate, estimate. The application of dual parameter analysis to sub-population quantitation needs further study.

### REFERENCES

Andreeff, M., Darzynkiewicz, Z., Sharpless, T. K., Clarkson, B. D. & Melamed, M. (1980) Discrimination of human leukaemia subtypes by flow cytometric analysis of cellular DNA and RNA. *Blood*, **55**, 282.

Barlogie, B., Hittelman, W., Spitzer, G. and 4 others (1977) Correlation of DNA distribution abnormalities with cytogenetic findings in human adult leukaemia and lymphoma. *Cancer Res.*, **37**, 4400.

Barlogie, G., Gohde, W., Johnston, D. A. & 4 others (1970) Determination of ploidy and proliferative characteristics of human solid tumours by pulse cytophotometry. *Cancer Res.*, **38**, 3333.

Braylan, R. C., Fowlkes, B. J., Jaffe, E. S., Sanders, S. K., Berard, C. W. & Herman, C. J. (1978) Cell volumes and DNA distributions of normal and neoplastic human lymphoid cells. *Cancer*, **41**, 201.

DIAMOND, L. W. & BRAYLAN, R. C. (1980) Flow analysis of DNA content and cell size in non-Hodgkin's lymphoma. *Cancer Res.*, **40**, 703.

LATREILLE, J., BARLOGIE, B., DOSIK, G., JOHNSTON, D. A., DREWINKO, B. & ALEXANIAN, R. (1980) Cellular DNA content as a marker of human multiple myeloma. *Blood*, **5**, 403.

MILTHORPE, B. K. (1980) FMFPAK1: A program package for routine analysis of single parameter flow microfluorimetric data on a low cost mini-computer. *Comp. Biomed. Res.* **13**, 417.

ROTMAN, B. & PAPERMASTER, B. W. (1966) Membrane properties of living mammalian cells as studied by enzymatic hydrolysis of fluorogenic esters. *Proc. Natl Acad. Sci., U.S.A.*, **55**, 134.

TAYLOR, I. W. & MILTHORPE, B. K. (1980) An evaluation of DNA fluorochromes, staining techniques and analysis for flow cytometry: 1. Unperturbed cell populations. *J. Histochem. Cytochem.* **28**, 1224.